# Pulmonary Inflammation of Well-Dispersed Multi-Wall Carbon Nanotubes Following Intratracheal Instillation: Toxicity by Fiber of 1–5 µm in Length

**DOI:** 10.3390/ma5122833

**Published:** 2012-12-13

**Authors:** Masanori Horie, Mayumi Stowe, Tatsunori Kambara, Byeong Woo Lee, Shigehisa Endoh, Junko Maru, Takako Oyabu, Toshihiko Myojo, Akira Ogami, Kunio Uchida, Kazuhiro Yamamoto, Norihiro Kobayashi, Estushi Kuroda, Tetsuya Nakazato, Yasuo Morimoto

**Affiliations:** 1 Institute of Industrial Ecological Sciences, University of Occupational and Environmental Health Japan, 1-1 Iseigaoka, Yahata-Nishi, Kitakyushu, Fukuoka 807-8555, Japan; E-Mails: msuto@med.uoeh-u.ac.jp (M.S.); 0015854@cc.m-kagaku.co.jp (T.K.); leebw401@med.uoeh-u.ac.jp (B.W.L.); toyabu@med.uoeh-u.ac.jp (T.O.); tmyojo@med.uoeh-u.ac.jp (T.M.); gamisan@med.uoeh-u.ac.jp (A.O.); yasuom@med.uoeh-u.ac.jp (Y.M.); 2 Research Institute for Environmental Management Technology (EMTECH), National Institute of Advanced Industrial Science and Technology (AIST), Tsukuba, Ibaraki 305-8565, Japan; E-Mails: s-endoh@tasc-nt.or.jp (S.E.); j_maru@tasc-nt.or.jp (J.M.); uchida-kunio@aist.go.jp (K.U.); tet.nakazato@aist.go.jp (T.N.); 3 Research Institute of Instrumentation Frontier (RIIF), AIST, 1-1-1, Umezono, Tsukuba, Ibaraki 305-8565, Japan; E-Mail: k-yamamoto@aist.go.jp; 4 National Institute of Health Sciences, Japan, Kamiyoga 1-18-1, Setagaya-ku, Tokyo 158-8501, Japan; E-Mail: norihiro.kobayashi@nihs.go.jp; 5 School of Medicine, University of Occupational and Environmental Health Japan, 1-1 Iseigaoka, Yahata-Nishi, Kitakyushu, Fukuoka 807-8555, Japan; E-Mail: kuroetu@ifrec.osaka-u.ac.jp

**Keywords:** multi-wall carbon nanotube, inflammation, chemokine, oxidative stress, fiber length

## Abstract

The pulmonary toxicity of multi-wall carbon nanotubes (MWCNT) were examined by intratracheal instillation. We prepared a well-dispersed MWCNT dispersion including MWCNTs of 3.71 µm geometric average length. The fiber length of most of the MWCNTs in the dispersion was 10 µm or less. The MWCNT dispersion was administered to rat lung by single intratracheal instillation at doses of 0.2 mg and 0.6 mg/rat. Bronchoalveolar lavage fluid (BALF) was collected at 3 days, 1 week, 1 month, 3 months, and 6 months after instillation. The influences of the longer MWCNTs on the induction of inflammation and oxidative stress were examined by the number of neutrophils, cytokine induced neutrophil chemoattractant-1 (CINC-1), CINC-2, CINC-3 and HO-1 in the BALF. Additionally, *ho-1* gene expression in the lung was examined. The intratracheal instillation of MWCNT induced transient inflammation dose dependently in the lung. The number of neutrophils was highest at 3 days after instillation and then decreased. However, the neutrophils in the MWCNT administered animals tended to be higher than in the control group until 3 months after instillation. The CINC-1 and CINC-2 concentrations in the BALF increased at 1 month after instillation. There were no significant differences in CINC-3 and HO-1 between the MWCNT administered animals and the control animals. These results revealed that the MWCNTs of 1–10 µm in length induced persistent inflammation in rat lung. There were no remarkable differences between the MWCNTs in the present study and previously reported, shorter MWCNTs prepared from “the same” raw MWCNT material.

## 1. Introduction

A carbon nanotube (CNT) is a nanomaterial consisting of only carbon. CNT has many beneficial features for industry, such as its rigidity and flexibility. CNT can be either a conductor or a semiconductor; that is determined by its chiral indices. Thus, CNT is an important functional material for industry. CNT is used in many industrial products, such as electronic devices, fuel cells and displays. Additionally, CNT is also investigated for life science, such as in drug delivery systems. Although CNT is a beneficial and important material, its harmful effects to apparatus respiratorius are feared [1]. There are some investigations about the pulmonary toxicity of CNT, demonstrating that it depends on the kind of CNT used for each examination because of the different responses of CNT. That is to say, the physical property of CNT influences its toxic activity [2]. Among the physical and chemical properties of CNT, it has recently been reported that the length of CNT fibers is related to its toxicity. In some *in vivo* and *in vitro* studies, long fibers (more than 10 µm in length) were pathogenic [3,4,5,6]. Intraperitoneal administration of two kinds of CNT of different lengths (<5 µm and >20 µm) to mice revealed granulomatous inflammation in animals that were administered longer CNT [7]. Liu *et al.* [5] reported in an *in vitro* study that longer (3–14 µm) multi-wall carbon nanotubes (MWCNTs) exerted high toxicity and that shorter MWCNTs were significantly less toxic. On the other hand, short fibers (less than 1 µm) of CNT have been reported to be more toxic than long fibers [6]. Short SWCNTs induced neutrophil infiltration in mice lung in an *in vivo* study [8] and apoptosis through reactive oxygen species (ROS) in an *in vitro* study, compared with longer fibers. It is therefore inconclusive whether the toxicity of CNTs depends on the length of fiber. Furthermore, the toxicity of CNTs with a length from 1 to 5 µm (intermediate length) has recently been reported. Manshian *et al.* [9] reported that among three different lengths of CNTs (400–800 nm, 1–3 µm and 5–30 µm), the intermediate type of CNTs (1–3 µm) induced the highest genotoxic potentials. Additionally, a CNT of 5 µm or less in length showed cytotoxicity [10]. It is important to estimate the pulmonary toxicity of intermediate length CNTs because of their direct exposure route. In the present study, we prepared individual MWCNTs, approximately 60% of whose length was 1 to 5 µm and 40% of whose length was 5–10 µm, and the effect of the fiber length on pulmonary inflammation was examined in an intratracheal instillation study.

## 2. Results

### 2.1. Characterization of MWCNT in the Dispersion

**Figure 1:**
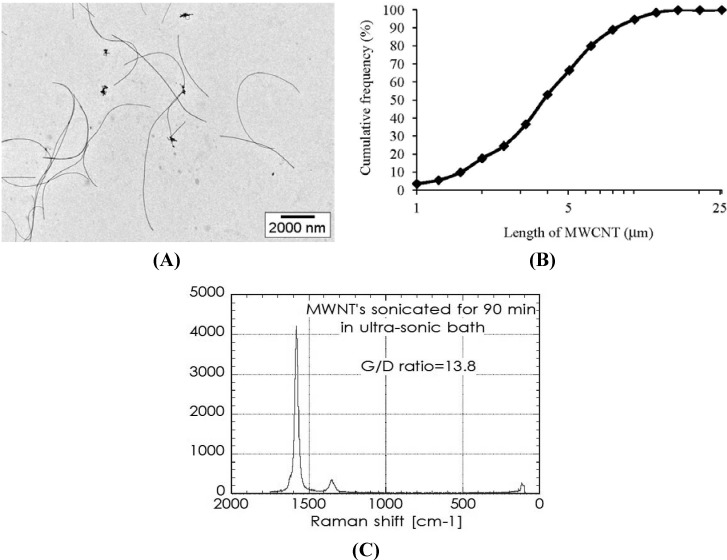
(**A**) Transmission electron microscopy (TEM) observation of multi-wall carbon nanotubes (MWCNTs) in the dispersion for intratracheal instillation; (**B**) Distribution of MWCNT length on the basis of TEM observation; (**C**) Raman spectra of the CNT in the dispersion.

The size distribution, Raman spectra and TEM images of MWCNTs in the dispersion for the intratracheal instillation are shown in Figure 1.

The geometric mean and geometric standard deviation were 3.71 µm and 1.93 µm, respectively. The median length was 3.67 µm and the G/D ratio was 13.8. Almost all of the MWCNTs were individually dispersed in the suspension. The percentage of those 1 to 5 µm in length was 60.38% as a distribution of numbers, and the percentage of those 5 to 10 µm in length was approximately 31%.

### 2.2. Analysis of Cells in BALF after Intratracheal Instillation

**Figure 2:**
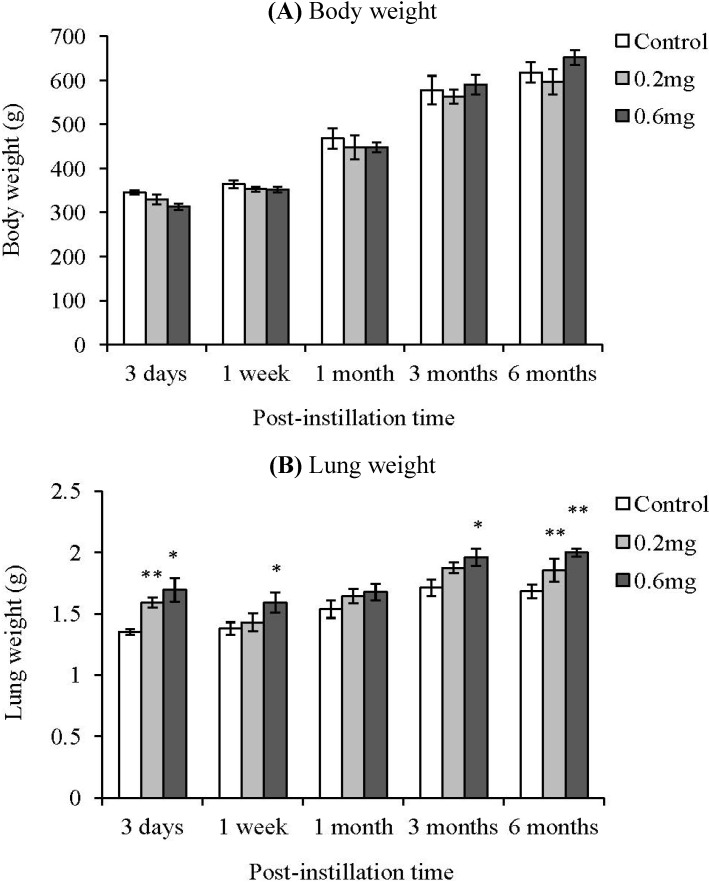
Body and lung weight of animals after intratracheal instillation of MWCNT. Values are mean ± SE (*n* = 5). *p < 0.05, **p < 0.01 (*vs.* each negative control group).

There was no significant difference in body weight between the control group and the MWCNT instillation groups (Figure 2). Compared with the control group, the lung weight in the MWCNT instillation groups tended to be heavy. There was a significant difference in lung weight between the control group and the MWCNT administered animals even at 6 months after intratracheal instillation. The total cell count in the BALF increased at 3 days after instillation in only the 0.6 mg-administered group.

**Figure 3:**
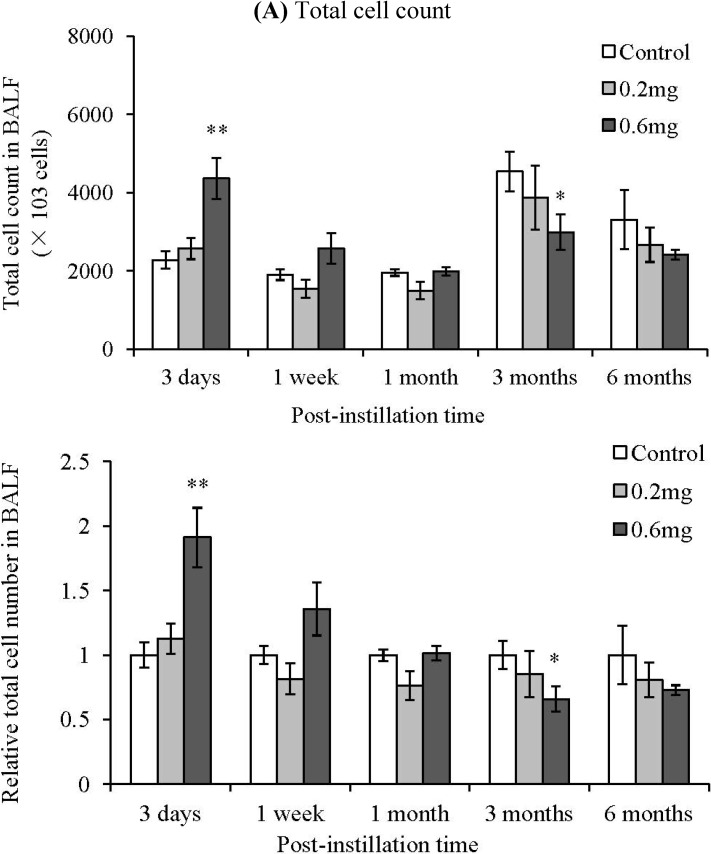

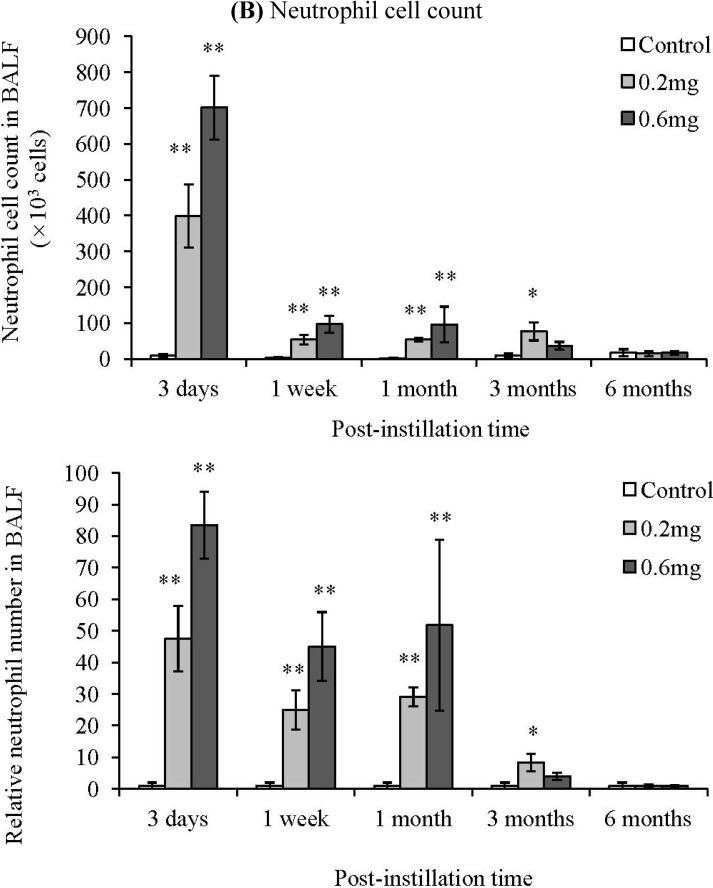
Bronchoalveolar lavage fluid (BALF) results after intratracheal instillation of MWCNT. (**A**) Total cell count; (**B**) Neutrophil cell count. Lower panels show relative level of the cell count in BALF. Values are mean ± SE (*n* = 5). *p < 0.05, **p < 0.01 (*vs.* each negative control group).

There was no significant difference between the control group and the MWCNT instillation groups from 1 week to 6 months after instillation (Figure 3A). Neutrophils significantly increased dose dependently at 3 days and 1 week after instillation of MWCNTs (Figure 3B). The relative level to control group of total cells and neutrophils in the BALF is also shown in Figure 3. Neutrophils in the BALF from the 0.2 mg-administered groups at 3 days and 1 week after instillation were 47 and 25 times higher than the control group, respectively. Neutrophils in the BALF from the 0.6 mg-administered groups at 3 days and 1 week after instillation were 84 and 45 times higher than the control group, respectively. At 1 month after instillation of MWCNTs, neutrophils in the BALF tended to increase, but there was no statistically significant difference because the neutrophil count varied widely among individual animals. Additionally, neutrophils significantly increased in the 0.2 mg-administered group at 3 months after instillation. At 6 months after instillation, there was no significant difference in the neutrophil count in the BALF between the control group and the MWCNT instillation groups.

### 2.3. Uptake of CNT by Macrophages

**Figure 4:**
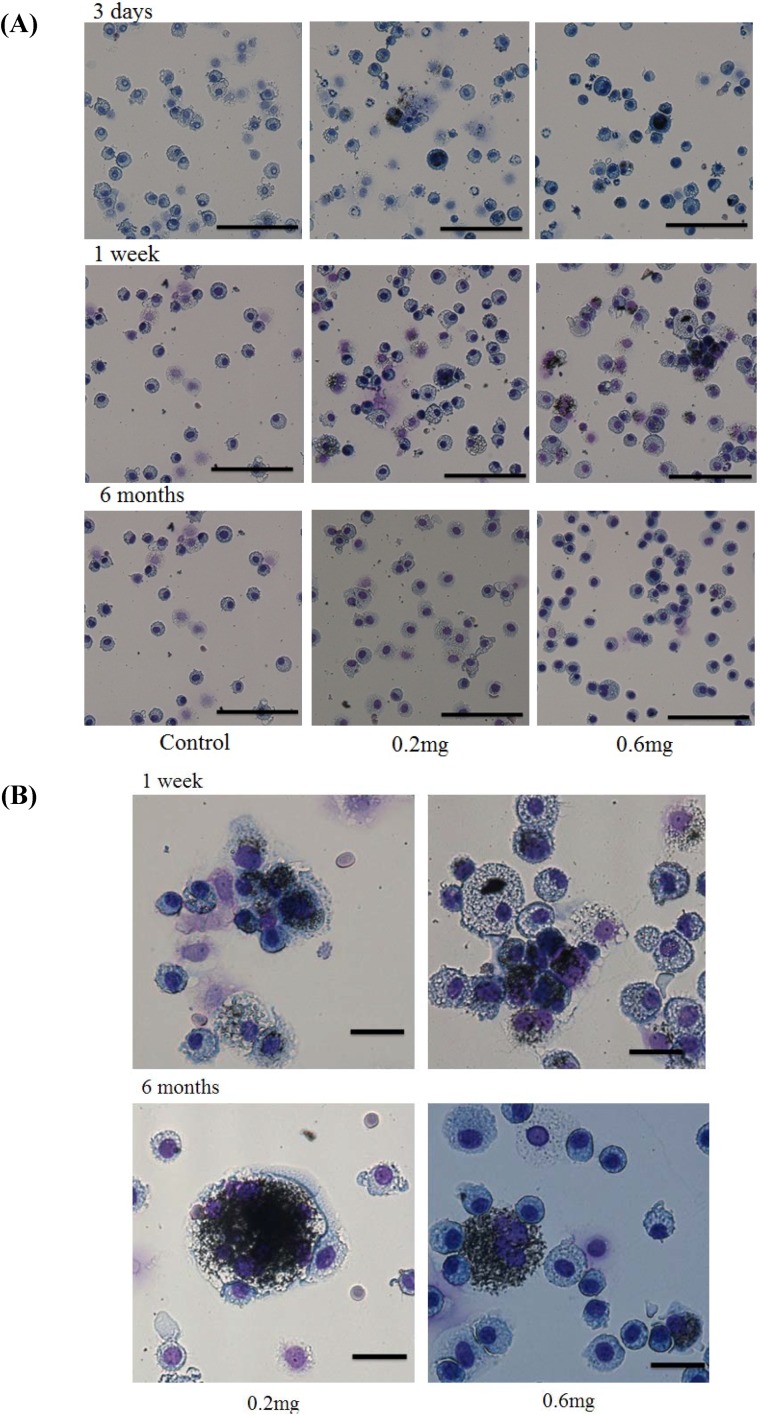
Observation of cells in the BALF and uptake of CNT by macrophages. (**A**) Light micrograph of total cells in the BALF. Bar = 100 µm; (**B**) Light micrograph of macrophage in the BALF at 3 days, 1week, and 6 months after instillation was shown. Bar = 20 µm. The cells in the BALF were stained by Diff-Quick stain. Phagocytosis of CNT by macrophage was observed.

Cells in the BALF at 3 days, 1 week and 6 months after instillation were observed. At 3 days and 1 week after instillation, many phagocytic macrophages were observed. Although the phagocytic macrophages decreased at 6 months after instillation, uptake of the CNT by macrophages was still observed at that time (Figure 4).

**Figure 5:**
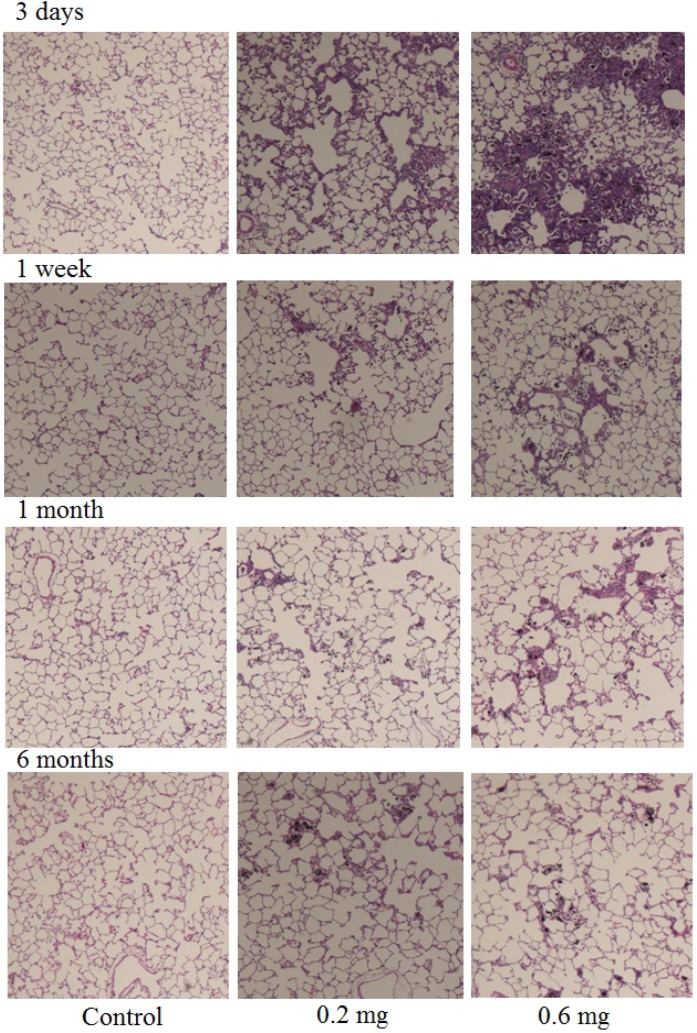
HE staining of lung sections exposed to MWCNT after intratracheal instillation. Control (0.05% of Triton X-100), low dose (0.2 mg) and high dose (0.6 mg) sections at 3 days, 1 week, 1 month, and 6 months after instillation were shown.

Hematoxylin-eosin (HE) staining of lung sections exposed to MWCNTs after intratracheal instillation is shown in Figure 5. In the present study, the MWCNT dispersion for intratracheal instillation was an isolated dispersion (Figure 1). However, internalized MWCNTs in the macrophages were observed as aggregates.

### 2.4. CINC Concentration in BALF

**Figure 6:**
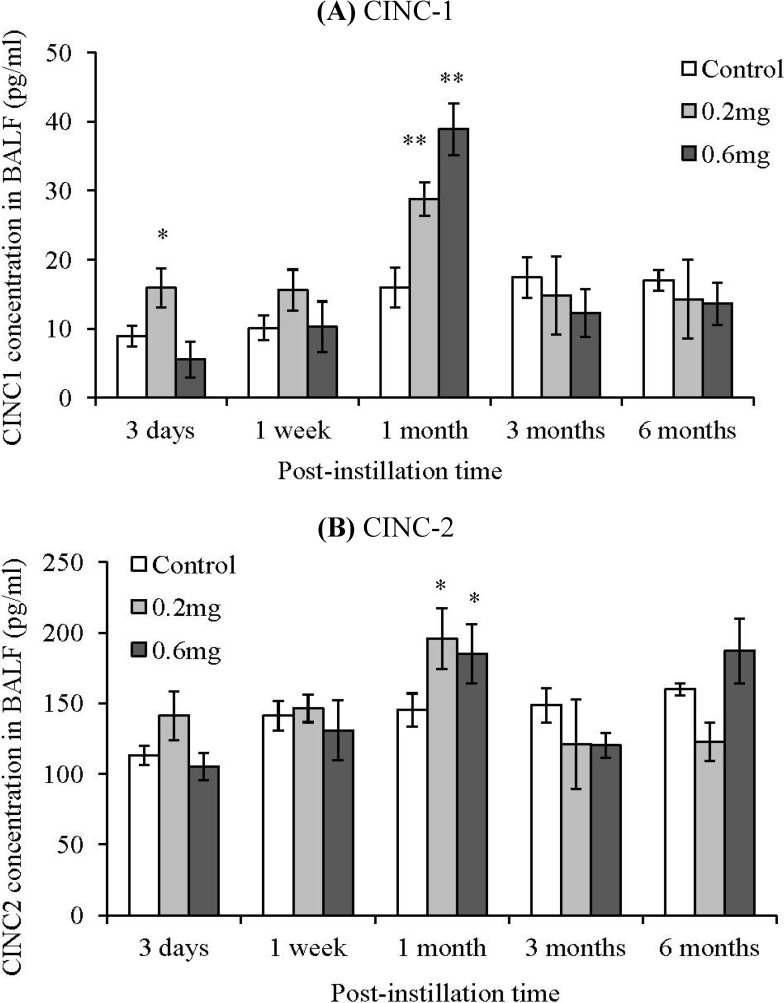
Concentration of CINC in BALF after intratracheal instillation of MWCNT. (**A**) CINC-1; (**B**) CINC-2. The concentrations of CINCs were measured by ELISA. Values are mean ± SE (*n* = 5). *p < 0.05, **p < 0.01 (*vs.* each negative control group).

The cytokine induced neutrophil chemoattractant (CINC) concentration in the BALF was measured in order to examine the inflammation response induced by the intratracheal instillation of MWCNTs (Figure 6). CINCs have a similar activity to IL-8; they promote the migration of neutrophils. The CINC-1 concentration in the BALF increased slightly at 3 days after instillation in the 0.2 mg-instillation group. At 1 month after instillation, the CINC-1 significantly increased dose dependently. CINC-2 also significantly increased dose dependently at 1 month after instillation. This increase of CINC-1 and CINC-2 was transient, and there were no significant differences between the control group and the MWCNT instillated groups at 3 months and 6 months after instillation. CINC-3 did not increase at any of the time points, and was below the detection limit.

### 2.5. HO-1 Concentration in BALF

**Figure 7:**
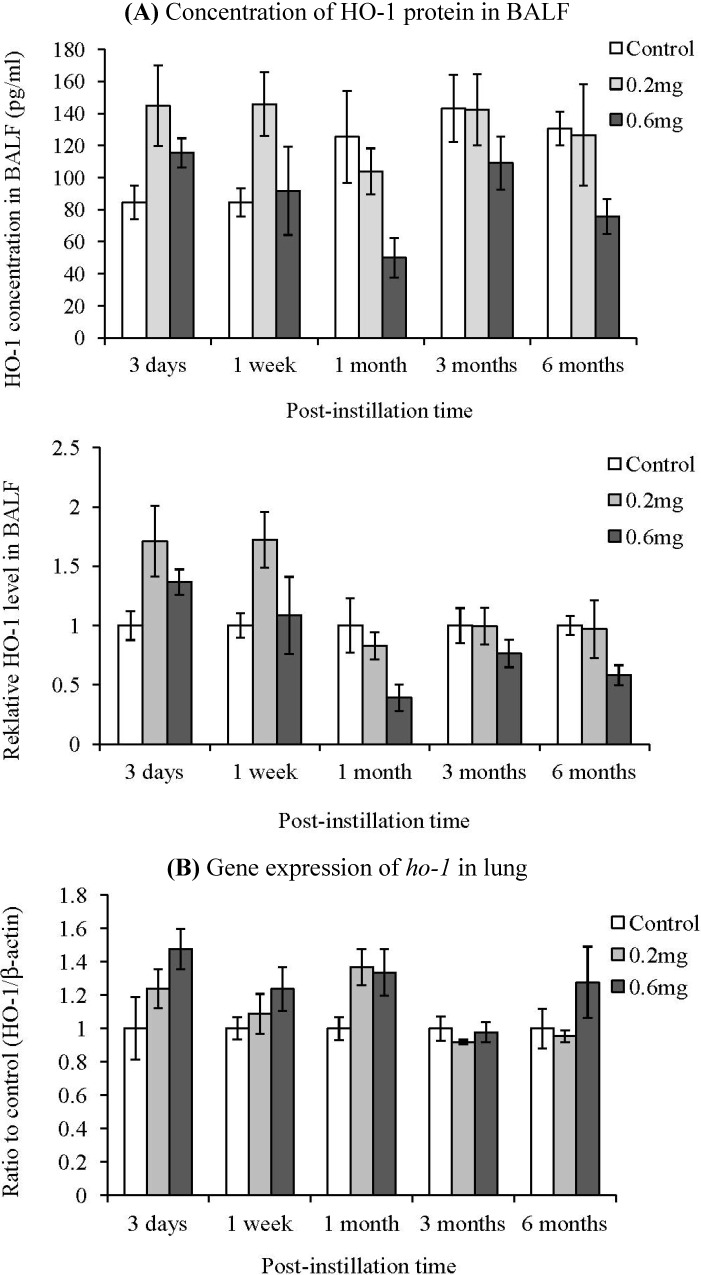
Influence of MWCNT on HO-1 expression after intratracheal instillation. Concentration of HO-1 protein in BALF. (**A**) The concentration of HO-1 was measured by ELISA. Lower panel shows relative level of the HO-1 protein in BALF; (**B**) Gene expression of *ho-1* in lung. The gene expression was detected by real-time PCR. Values are mean ± SE (*n* = 5).

The HO-1 concentration in the BALF and *ho-1* gene expression in lung were measured in order to examine the induction of oxidative stress by the intratracheal instillation of MWCNTs (Figure 7). Relative level to control group of HO-1 in the BALF is also shown in Figure 6. The HO-1 levels in the BALF from the 0.2 mg-administered groups at 3 days and 1 week after instillation were 1.7 times higher than the control group. Neutrophils in the BALF from the 0.6 mg-administered groups at 3 days and 1 week after instillation were 0.4 and 1.1 times higher than the control group, respectively. Although the HO-1 concentration in the BALF tended to increase at 3 days after instillation, there were no significant differences between the control group and the MWCNT instillated groups at any of the time points. The gene expression of *ho-1* in the lung did not increase, either.

## 3. Experimental Section

### 3.1. MWCNTs

MWCNTs were provided by Nikkiso Co., Ltd, Tokyo, Japan. The MWCNTs were synthesized by the catalytic chemical vapor deposition (CVD) method. The raw MWCNT was the same as the material used in our previous study [11]. The characteristics of the raw MWCNTs were as follows: Diameter was 44 nm, BET surface area was 69 ± 37 m^2^/g. Metal content was measured by inductively coupled plasma-mass spectrometry (ICP-MS) analysis. The results were as follows: Li 0.5 μg/g, Al μg/g, Ca 176 μg/g, Fe 53 μg/g, and Cd 16 μg/g.

### 3.2. Animals

Male Wistar rats (8 weeks old) were purchased from Kyudo Co., Ltd (Kumamoto, Japan). The animals were kept in the Laboratory Animal Research Center of the University of Occupational and Environmental Health for a week with free access to commercial diet and water. All procedures and animal handling were performed according to the guidelines described in the Japanese Guide for the Care and Use of Laboratory Animals as approved by the Animal Care and Use Committee, University of Occupational and Environmental Health, Japan.

### 3.3. Preparation of MWCNT Dispersions

The MWCNTs (Nikkiso Co., Japan) used in the instillation tests were dispersed into water by sonication using an ultrasonic bath. 50 mg of the MWCNTs was placed in a 100 mL vial, and 50 mL of Milli-Q water (Millipore, USA) including Triton X-100 (Wako, Japan) of 0.5 mg/mL as a dispersant was added to the vial. Sonication to the vial was carried out for 90 min with an ultrasonic bath (BRANSON 5510, Branson, USA; 135 W at a frequency of 42,000/s). During the dispersion treatment, the water level in the bath and the position of the vial were tuned so that the sonication worked under resonance conditions. The CNT suspension was filtered through a sieve with 50 µm apertures to remove flocs. The concentration of CNTs in the suspension was 1.35 mg/mL, and the suspension was diluted by Milli-Q water to adjust the concentration. CNT suspensions with different concentrations of CNT were used for the intratracheal instillation.

### 3.4. Characterization of MWCNT

For the raw MWCNTs and dispersed MWCNTs in the dispersion, tube morphology was evaluated on the basis of observations using a transmission electron microscope (TEM; JEM-1010; Jeol Ltd., Tokyo, Japan) at the acceleration voltage of 100 kV. In order to evaluate the degree of MWCNT damage to the sample preparation, the presence of defects in the grapheme structure of the raw MWCNTs and the MWCNT dispersions was evaluated by Raman spectroscopy (Nicolet Almega XR micro-Raman system; Thermo Fisher Scientific Inc., Japan). The resonance Raman scattering spectra were measured in the frequency regions of 100–1800/cm with an excitation wavelength of 532 nm.

### 3.5. Intratracheal Instillation of MWCNT

All animal experiments were approved by the Institutional Animal Care and Use Committee (IACUC) of the University of Occupational and Environmental Health, Japan. A total of 0.2 mg (0.66 mg/kg) or 0.6 mg (1.98 mg/kg) of MWCNTs was dispersed in 0.4 mL of distilled water including 0.05% Triton X-100 as a dispersant. Each material dispersion was intratracheally instilled once in Wistar male rats (9 weeks old). The negative control group was exposed to distilled water including 0.05% Triton X-100. The animals were dissected at 3 days, 1 week, 1 month, 3 months and 6 months after instillation. Each group consisted of 10 animals and was divided into two subgroups for lung tissue analysis. The first subgroup (five rats) provided bronchoalveolar lavage, which was collected using physiological saline that was injected through a cannula inserted in the respiratory tract, into the right lung, while the left lung was clamped. Three to 10 mL (different volumes of lavage fluid were based on animal ages) of physiological saline was infused in the right lung each time, and up to 50 mL lavage fluid was collected in total. The lungs of the second subgroup (five rats) were homogenized to extract mRNA.

### 3.6. Observation of Cells in the BALF

The cells were collected from the BALF by centrifugation at 1500 rpm for 15 min. The cells were then washed once with a PMN buffer (4.03 g of NaCl, 0.1 g of KCl, 0.58 g of Na_2_HPO_4_, 0.1 g of KH_2_PO_4_, 0.5 g of glucose in 500 mL of distilled water) and finally were resuspended in the PMN buffer at a concentration of 2 × 10^5^ cells/mL. The slide was prepared by Auto Smear (Sakura Seiki Co., Ltd., Tokyo, Japan). The cells were then stained by Diff-Quik (Sysmex Corporation, Kobe, Japan) and observed by a light microscope (Olympus BX50, Olympus Corporation, Tokyo, Japan).

### 3.7. Measurement of Chemokines and HO-1 in the BALF

The concentrations of cytokine induced neutrophil chemoattractant-1 (CINC-1), CINC-2, and CINC-3 in the BALF were measured by enzyme linked immunosorbent assay (ELISA) using Quantikine Rat CINC-1, CINC-2, and CINC-3 (R&D Systems, Inc., Minneapolis, MN, USA). The HO-1 concentration was determined by ELISA using a HO-1 (rat) ELISA kit (ENZO Life Sciences, Inc., Farmingdale, NY, USA).

### 3.8. Detection of Gene Expression of *ho-1* in Lung

The expression of the *ho-1* gene was determined by real time polymerase chain reaction (Real time-PCR). Total RNA was isolated from the cells using the RNeasy protect mini kit (Qiagen GmbH, Hilden, Germany). cDNA synthesis was carried out with a High Capacity cDNA Reverse Transcription kit (Applied Biosystems, Carlsbad, CA). Real time-PCR was conducted by a Step One real time-PCR system (Applied Biosystems), and PCR amplification of the lung tissues was detected by TaqMan^®^ gene expression assays (Applied Biosystems). Rat β-actin gene was used as an endogenous control. The assay ID of TaqMan^®^ gene expression assay for rat heme oxygenase-1 (HO-1) was Rn00561387_m1 (Applied Biosystems). The gene expression level was analyzed by the relative standard curve method.

### 3.9. Tissue Preparation for Hematoxylin-Eosin Staining

The lung (total left lobe), which was inflated and fixed by 4% paraformaldehyde, and the trachea were resected from the surrounding tissue. The lung tissue was embedded in paraffin and 5 µm thick sections were cut from the lobe. The samples were then sectioned and stained with HE.

### 3.10. Statistical Analysis

Data are means ± S.D. of at least three separate experiments. Statistical analyses were carried out by the Mann-Whitney U test.

## 4. Discussion

Recent investigations suggest that fiber length is one of the important factors in the pulmonary toxicity of CNTs. The mean length of the MWCNTs used in the present study was 3.71 µm, and the percentage of those 1 to 5 µm in length was 60.38%. The percentage of the CNT fibers whose length was 10 µm or less was approximately 95%. There is a report that CNTs with a fiber length of 1–5 µm show stronger toxic activity than other lengths [8]. In the present study, a persistent inflammation increase was observed by intratracheal instillation of MWCNTs whose length was approximately 1–10 µm of which 60% were 1–5 µm. According to an examination of the pharyngeal aspiration of short (range 1–5 µm) or long (mean 13 µm) MWCNTs to mice, the short MWCNTs caused a greater increase of neutrophils in BALF than did the long MWCNTs [8]. Our result in the present study that MWCNTs caused inflammation conforms to these previous studies. On the other hand, it has been reported that pulmonary inflammation caused by long fiber CNTs was rapidly recovered from [4]. The retention in the lung of inhaled fibers is related to their pulmonary toxicity [12]. In the present study, neutrophils in the BALF remarkably increased by intratracheal instillation of MWCNTs at 3 days after instillation, after which the number of neutrophils decreased at 1 week after instillation. The cause of the rapid recovery from pulmonary inflammation has been considered to be the low retention of CNTs in the lung. However, the neutrophil count in the BALF from the MWCNT instillation groups did not recover to control level even 3 months after instillation. Thus, in the present study, the pulmonary inflammation persisted, suggesting the possibility of low clearance of MWCNT fiber from the lung. Actually, MWCNTs phagocytized by pulmonary macrophages were still observed 6 months after instillation. There is also a report that the fiber length of CNTs was related to pleural translocation of isolated tubes [4]. The pleural translocation of isolated tubes was observed in long CNTs whose mean length was 36 µm. Short CNTs whose length was 1–5 mm did not show pleural translocation. On the other hand, pleural penetration of MWCNTs whose median length was 3.86 µm was observed after pharyngeal aspiration in mouse [13]. Mercer *et al.* reported that 60% of MWCNTs were ingested by macrophages at 1 day after aspiration. Phagocytosis of the MWCNTs by macrophages was also observed in the present study. Phagocytosis by macrophages is important in the pulmonary toxicity of MWCNT. Compared with a short MWCNT, the phagocytosis of a longer MWCNT by macrophages is difficult and thus it may show larger pulmonary toxicity. Additionally, overload of an instillation material which exceeds the phagocytic capacity of the macrophage leads to delay of clearance [14]. Overload induces inflammation by even low toxic materials such as TiO_2_. According to an inhalation test on TiO_2_ to rodents, overload was shown at a dose of 10 mg/m^3^ [14]. In the present study, persistent inflammation was observed in not only the high dose group but also in the low dose group. The overload could not have been the cause of the persistent inflammation induced by the intratracheal instillation of the MWCNTs.

Although the MWCNT in the dispersion was an isolated dispersion, internalized MWCNT in the macrophage was observed as aggregates (Figure 4). In intratracheal instillations, the dispersion state of the CNT is very important. Well-dispersed CNT reaches the pulmonary alveolus. However, large aggregates of CNT cannot reach the pulmonary alveolus; thus, there are different biological responses between the well-dispersed CNT and the aggregated CNT. Pharyngeal aspiration of agglomerate SWCNT to mouse induced granulomatous lesions. However, more dispersed SWCNT did not induce granulomatous lesions [15]. In the present study, we could not find any pleural translocation of MWCNT.

CNT adsorbs biological molecules such as proteins [16]. A CNT which reaches the pulmonary alveolus as an isolated dispersion will adsorb biomolecules such as surfactant proteins and lipids. Similarly, a CNT that reaches the pulmonary alveolus by inhalation will also adsorb biomolecules onto the surface, and then the CNTs may form an aggregate/agglomerate at the pulmonary alveolus by interaction with the biomolecules. Moreover, there is also the possibility that MWCNTs form aggregates after phagocytosis in the macrophage. In the next stage, it will be necessary to examine the influence of reaggregation of CNTs on phagocytosis of the macrophages in the pulmonary alveolus.

We previously reported that an increase of inflammation at the acute phases in rat was shown by intratracheal instillation of a MWCNT that was shorter than the one used in the present study [11]. In the present study, we used the same MWCNT material as in the previous study and prepared a MWCNT dispersion that included longer MWCNT fibers (3.71 µm) than in the previous study (0.94 µm). The fiber length of the MWCNTs used in the present study was approximately four times longer, on geometric average, than in the previous one. These two experiments used the same MWCNT material; therefore, the diameter was also the same. Both the experiments showed persistent inflammation from the acute phase to the chronic phase. Although these experiments cannot be compared directly because they were not performed simultaneously, these results suggest that the relationship between inflammation and the fiber length of CNTs is small in this region of fiber length (5 µm or less). The measurement of the CINC family, which is one of the chemokines against neutrophil in the BALF, showed only a transient increase of CINC-1 and CINC-2, while CINC-3 did not increase at any time point. In many cases, secretion of the CINC family is related to inflammation. It has been reported that hazardous particles such as crystalline silica and diesel exhaust particles induced inflammation with an increase in the CINC family [17,18], and TiO_2_ particles, which are known as low-toxic particles, do not show an induction of the CINC family [19]. Our previous study using shorter MWCNTs showed continuous inflammation and CINC-1 expression in the high dose group. In contrast, although neutrophil infiltration was observed until 3 months after instillation in the present study, the expression of CINC was transient.

Although the reason for the different dynamic states between the inflammation and the CINC expression in this study is unclear, some possibilities about the causes can be considered. The increase of neutrophils in the BALF was small in the chronic phase, thus the mild inflammation did not lead to an expression of CINC. It has also been reported that other chemokines, such as GCP-2, are related to neutrophil-related inflammation [20]. There is a possibility that another chemokine, such as MIP-1, was related to the inflammation. Additionally, the administered dose might affect the expression of CINC. When continuous CINC-1 expression was observed in our previous study, the dose of MWCNT was 1.0 mg/animal. On the other hand, the “high dose” in the present study was 0.6 mg/animal.

Moreover, no expression of HO-1 was observed in the present study. Gene expression of HO-1 is upregulated by various stresses, including oxidative stress, such as free radical generation, and HO-1 reduces oxidative stress and oxidative stress-related inflammation [21,22,23]. In many cases, oxidative stress is related to inflammation caused by inhalation of particles [24,25,26]. It has also been reported that SWCNTs whose fiber length was 1–3 mm showed stronger ROS generation in culture cells than longer SWCNTs [9]. Exposure to inflammation inducible materials, such as crystalline silica and asbestos, induced oxidative stress and enhanced the expression of HO-1 in lung [27,28]. Therefore, there is a possibility that oxidative stress was not related to the inflammation caused by the MWCNTs in this study.

The results of the present study suggest that intratracheal instillation of MWCNTs induced persistent inflammation in lung, and that oxidative stress was not involved in the pulmonary toxicity of the MWCNTs. According to the present and our previous study, the effect of fiber length of CNTs on pulmonary toxicity is small, at least in the range of 0.94 to 3.7 µm in length. Compared with 0.94 µm MWCNT, 3.71 µm MWCNT did not cause severe pulmonary toxicity.
